# Real-Time Perfusion Assessment with Hyperspectral Imaging After Revascularization in Peripheral Artery Disease

**DOI:** 10.3390/jcm15041667

**Published:** 2026-02-23

**Authors:** Michaela Kluckner, Wolfgang Hitzl, Florian K. Enzmann, David Wippel, Maximilian Lutz, Sabine Wipper

**Affiliations:** 1 Department of Vascular Surgery, Medical University Innsbruck, 6020 Innsbruck, Austria; 2 Research and Innovation Management (RIM), Team Biostatistics and Publication of Clinical Trial Studies, Paracelsus Medical University, 5020 Salzburg, Austria; 3 Department of Ophthalmology and Optometry, Paracelsus Medical University Salzburg, 5020 Salzburg, Austria; 4 Research Program Experimental Ophthalmology and Glaucoma Research, Paracelsus Medical University, 5020 Salzburg, Austria; 5 Department of Radiology, Medical University Innsbruck, 6020 Innsbruck, Austria

**Keywords:** peripheral arterial disease, microperfusion, quantification, hyperspectral imaging, transcutaneous oxygen pressure measurement

## Abstract

**Background/Objectives**: Hyperspectral imaging (HSI) facilitates noninvasive assessment of tissue perfusion in patients with peripheral arterial disease. However, available studies are either based on small cohorts and provide no comparison to standard methods or only one-time measurements. **Methods**: In this prospective cohort study, assessment of tissue perfusion with transcutaneous oxygen pressure (TcPO2) measurement and HSI before (1 day) and after revascularization (1–3 days) in patients with Rutherford category 3–6 was performed. The primary endpoint was change in tissue perfusion evaluated with the different methods. HSI and TcPO2 were correlated with clinical improvement after revascularization. **Results**: Significant improvement in the perfusion was detected by tissue oxygenation in the microcirculation (StO2; improvement +12%, mean difference 5 ± 15.9, *p* < 0.001) and near-infrared spectroscopy (NIR; improvement +9%, mean difference 3.7 ± 7.1, *p* < 0.001), but not with the tissue hemoglobin index (THI; mean difference +0.8 ± 10.3, *p* = 0.428). A high number of worse or unchanged HSI measurements despite successful revascularization was detected. A significant improvement of TcPO2 after revascularization (mean difference +16.2 ± 27.7 mmHg, *p* < 0.001), consistent with clinical improvement, was detected. No correlation of the HSI parameters with TcPO2 or clinical symptoms could be seen. **Conclusions**: Significant improvement of StO2, NIR and TcPO2 values was detected after successful revascularization; however, no correlation of HSI parameters with TcPO2 or clinical results could be observed. Furthermore, the substantial rate of lower or unchanged HSI parameters despite clinical improvement and higher TcPO2 values calls the validity and clinical relevance of TIVITA^®^-based HSI measurements for postoperative tissue perfusion improvement into question.

## 1. Introduction

With the increased life expectancy of the population, the number of patients with peripheral arterial disease (PAD) is steadily rising. Due to this development, PAD patients also ‘live to experience’ the progression of their disease into chronic limb-threatening ischemia (CLTI) [1]. Assessment of tissue perfusion is crucial for grading the severity of CLTI as well as the prospects of wound healing in case of tissue loss [2]. The measurement of transcutaneous oxygen pressure (TcPO2) is one of the most commonly used methods in clinical practice for the evaluation of tissue perfusion. Multiple studies, including systematic reviews, indicate that TcPO2 values above established thresholds are associated with higher wound-healing rates, whereas lower values predict poorer healing and a greater risk of complications such as delayed or non-healing and subsequent amputation [3,4,5].

Hyperspectral imaging (HSI) is based on the principle of imaging reflectance spectroscopy with rapidly growing applications in the military, industrial and medical fields [6]. This noninvasive optical modality acquires spatially resolved spectral information across a broad range of visible and invisible wavelengths, enabling the characterization of tissue-specific absorption and scattering properties. By exploiting these spectral signatures, HSI facilitates the assessment of physiological parameters such as tissue oxygenation and perfusion [7]. Each measurement takes around five to six seconds without special instructions for use and can be performed in an outpatient setting as well as intraoperatively. Despite the relative novelty of this technology, the applicability has been tested in different clinical settings, including retinal eye disease, cancer detection and therapy, wound healing in diabetic foot ulcers and PAD. Grambow et al. showed that HSI is robust against confounders like ambient temperature and physical activity as well as a high interoperator reliability [8]. These results underscore the broad applicability of HSI across diverse clinical settings without the need for special environmental requirements, thereby facilitating its routine clinical use. Chiang et al. evaluated HSI in the diagnostics of PAD with a correlation of established measures, including TcPO2, ABI, skin temperature, and clinical severity. The authors found that hyperspectral parameters, particularly oxygen saturation, showed strong intra- and interoperator reliability as well as significant correlations with disease severity and other physiological markers. The results suggest that HSI is a reliable, rapid, noninvasive tool with potential value as an early screening method for PAD [9]. Kleiss et al. assessed tissue perfusion with HSI and thermal imaging before and after endovascular revascularization for PAD. In this small study with 23 patients and 29 investigated limbs, HSI was able to detect changes in tissue perfusion. Deoxyhemoglobin was also significantly different in patients with and without clinical improvement during follow-up [10]. Oxy- and deoxyhemoglobin levels as well as oxygen saturation were measured before and after endovascular and surgical revascularization by van Schilt et al. After improvement of perfusion, a significant decrease in deoxyhemoglobin and an increase in oxygen saturation were detected [11]. Both studies included only small patient cohorts and did not incorporate a comparison with established perfusion assessment methods. Most available studies dealing with HSI for perfusion assessment in PAD lacked any comparison with standard techniques for tissue perfusion measurement, included only one HSI measurement, or had relatively small study cohorts [6,12,13,14,15].

The aim of this prospective validation study was to determine whether TIVITA^®^-based HSI parameters correlated with TcPO2 parameters as well as with clinical improvement following successful revascularization.

## 2. Materials and Methods

Patients with Rutherford categories 3 to 6 undergoing revascularization at a university vascular center were prospectively enrolled in this study. Enrollment started in October 2022 and ended in February 2025. Follow-up visits were held three months after the revascularization; the last follow-up visit occurred in May 2025. No further data was obtained after the final visit.

Patients with ulcerations prior to revascularization were further categorized applying the WIfI classification [2]. At the predefined measurement site between the first and second metatarsal bones of the treated limb, tissue perfusion was assessed with one measurement using TcPO2 and HSI, respectively, before and after technically successful revascularization. Technical success was defined as completion of the procedure and <30% residual stenosis. In patients undergoing endovascular revascularization, measurements were performed on the second post-interventional day prior to discharge, whereas in surgically treated patients, measurements were obtained between postoperative days two and three.

**Figure 1:**
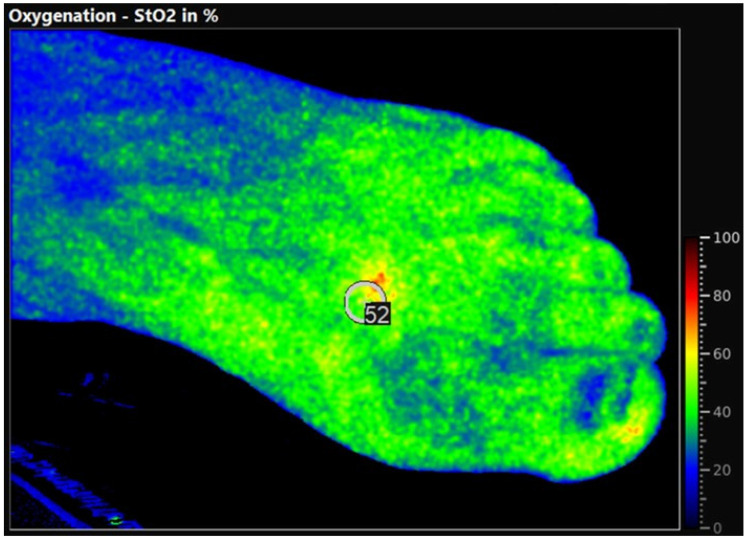
StO2 measurements with hyperspectral imaging. The marker for measurement was placed between metatarsal bones I and II.

The perfusion measurements were obtained in a supine position without room darkening or special room temperature control according to the IFU of the manufacturer. For the HSI measurement, the TIVITA^®^ 2.0 Surgery Edition (Diaspective Vision GmbH, Am Salzhaff, Germany) was used. The distance and field of view were predefined by the camera system by laser markings of the camera. The marker size was adapted to the diameter of the TcPO2 probe (1.5 cm). Three HSI parameters were analyzed in this study: (1) tissue oxygenation in the microcirculation (StO2): reflects the percentage of hemoglobin oxygenation in the capillary area of the tissue microcirculation and shows the changes in oxygen supply and consumption directly in the tissue; (2) the tissue hemoglobin index (THI); and (3) tissue oxygenation measurement with near-infrared spectroscopy (NIR): provides values for local hemoglobin saturation at the measurement site (Figure 1). The ankle–brachial index (ABI) was additionally measured pre- and post-procedural to verify the technical success of the revascularization. In case of non-measurable ABI due to media sclerosis, clinical improvement as well as a duplex ultrasound confirmed the technical success of the revascularization. The TcPO_2_ sensor was placed at the defined study location, measuring the pressure for 20 min in a supine position.

Chronic kidney disease with a glomerular filtration rate <30 mL/min, hyperthyroidism, patients unable to give informed consent, pregnancy, ulceration at the predefined measurement site and gangrene/active inflammation of the foot were the exclusion criteria for this study. Furthermore, patients with unsuccessful revascularization were also excluded from this study. Clinical follow-up was performed three months after the revascularization according to the routine follow-up regime of the department, including clinical examination, ABI-measurement and duplex ultrasound. This study was approved by the local ethics committee (EK Nr: 1440/2021) and registered at ClinicalTrials.gov (NCT05570019).

The primary endpoint of this study was the HSI parameter (StO2, THI, NIR) change after successful revascularization. This change was correlated with the improvement of perfusion measured with TcPO2 and ABI. A correlation of the clinical follow-up three months after the revascularization with HSI- and TcPO2 measurements was defined as the secondary endpoint of this study.

### Statistical Analysis

Based on an a priori power calculation, a sample size of 168 patients with an assumed dropout rate of 10% was planned. Although no formal interim analysis was pre-specified, a pragmatic interim review was performed during the course of this study. After review of the accumulated data, the study team decided to discontinue recruitment after 109 patients due to loss of clinical equipoise and futility, as continuation was considered unlikely to alter the overall conclusion.

Data were checked for consistency and normality using the Shapiro–Wilk test. In case of normal distributions, independent and dependent *t*-tests were used; otherwise, bootstrap-t tests based on 5000 Monte Carlo simulations were applied to compare means illustrated using whisker plots. For non-normally distributed data, a paired Wilcoxon test was used. Spearman correlations were computed and tested. All reported tests were two-sided, and *p*-values < 0.05 were considered statistically significant. All statistical analyses in this report were performed by use of STATISTICA 13 (Cloud Software Group, Inc., Data Science Workbench, version 14., San Ramon, CA, USA) and PASW 27 (IBM SPSS Statistics for Windows, Version 27.0., Armonk, NY, USA).

## 3. Results

Between October 2022 and February 2025, 109 patients were prospectively enrolled in this study; however, after the revascularization, two patients withdrew their consent. Four patients had to be excluded due to technical problems with HSI data storage. The final analysis included 103 patients. An overview of the demographic data is presented in Table 1. They reflect the typical risk factors and comorbidities of a PAD cohort with hypertension (77%), hyperlipidemia (56%), diabetes (35%), coronary artery disease (46%) and 71% former or active smoking status. Most patients presented with intermittent claudication as the leading symptom (69% Rutherford 3). Of the 32 (31%) CLTI patients, 12 (12%) presented with tissue loss. These patients were categorized at WIfI stage 4 in six cases, stage 3 in five cases and stage 1 in one patient.

An endovascular revascularization was performed in 54 (52%) patients, followed by surgical (n = 28, 27%) and hybrid procedures (n = 21, 20%). In the endo group, the femoropopliteal segment (59%) and the iliac (41%) arteries were the most commonly treated vessels. In the surgical group, patch plasty of the common femoral artery (43%) or femoropopliteal bypass (36%) was performed. All procedures were technically successful and documented with digital subtraction angiography. No bleeding complications or reinterventions for early reocclusion were necessary. A superficial wound-healing disorder in the groin was treated in one patient in our outpatient clinic; however, no deep surgical site or graft infections occurred in the postoperative course.

### 3.1. Perfusion Measurements

Due to media sclerosis or severely impaired perfusion, no ABI could be measured in 20 (19%) patients before the revascularization. After the procedure, no measurement was possible in four (4%) of these patients. The ABI significantly improved by a mean of 0.42 ± 0.3 (*p* < 0.001). The mean TcPO2 value prior to revascularization was 41.5 ± 22.8 mmHg with an improvement of 39% after the revascularization, resulting in a mean value of 57.7 ± 25.6 mmHg (mean difference 16.2 ± 27.7 mmHg, *p* < 0.001). In eight (8%) patients, no change in TcPO2 value was measured; in 24 (23%), the measurement was worse after the revascularization.

**Figure 2:**
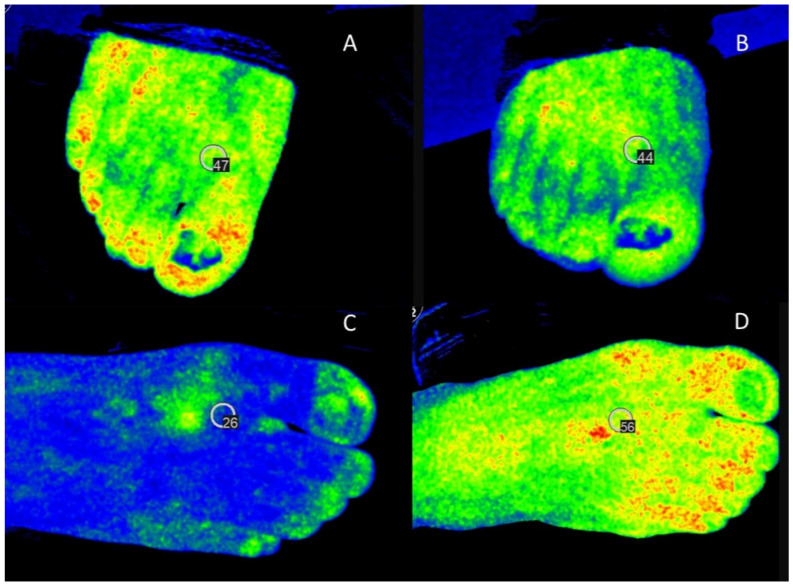
HSI StO2 measurements of a patient with worse measurements despite successful revascularization ((**A**) before, (**B**) after) as well as of a patient with improved results after the revascularization ((**C**) before, (**D**) after).

Regarding HSI, the mean StO2 improved by 12% (mean difference 5 ± 15.9, *p* < 0.001); however, in 34 (33%) patients worse and in six (6%) patients equal values were detected after successful revascularization (Figure 2).

**Figure 3:**
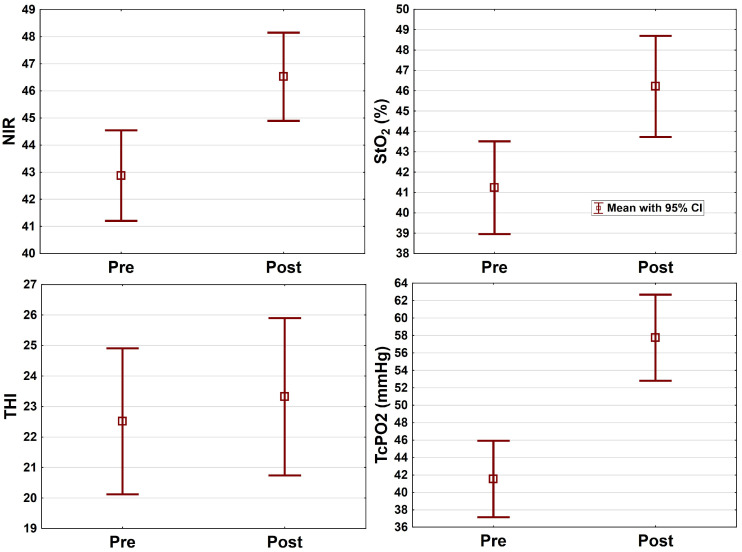
Boxplots of HSI (NIR, StO_2_, THI) and TcPO2 measurements before and after the revascularization, n = 103. NIR: tissue oxygenation measurement with near-infrared spectroscopy; StO2: tissue oxygenation; THI: tissue hemoglobin index; TcPO2: transcutaneous oxygen pressure measurement. Pre: measurement before the revascularization. Post: measurement after the revascularization.

The mean NIR before revascularization was 42.9 ± 8.7, followed by 46.5 ± 8.4, resulting in an improvement of 9% (mean difference 3.7 ± 7.1, *p* < 0.001). Despite successful revascularization, worse results were detected in 28 (27%) and stable results in 12 (12%) patients. THI showed no improvement after the revascularization, with mean values of 22.5 ± 12.4 and 23.3 ± 13.3, respectively (mean difference 0.8 ± 10.3, *p* = 0.428). Fifty-one (50%) patients had worse and nine (10%) patients had unchanged THI values despite successful revascularization. An overview of the mean TcPO2 and HSI values before and after revascularization is provided in Figure 3.

A significant, positive correlation between the improvement of ABI and TcPO2 values (r = 0.2436, 95% CI: 0.047–0.42, *p* = 0.0141) as well as ABI and NIR values (r = 0.22, 95% CI: 0.025–0.40, *p* = 0.029) could be observed. No significant association of other HSI parameters and ABI (StO2 *p* = 0.22, THI *p* = 0.22) or TcPO2 improvement and the three HSI parameters (StO2 *p* = 0.53, THI *p* = 0.68, NIR *p* = 0.89) was detected.

Subgroup analyses were performed to examine the influence of revascularization strategy and the Rutherford category at intervention on pre- and post-revascularization perfusion parameters. A significant difference in pre-revascularization mean STO2, ABI and TcPO2 values of patients undergoing endovascular or open/hybrid procedures could be observed (StO2 endo 37.4 ± 11.2 vs. open/hybrid 45.8 ± 11.4, *p* = 0.001; ABI endo 0.54 ± 0.16 vs. open/hybrid 0.44 ± 0.16, *p* = 0.003; TcPO2 endo 50.3 ± 19.2 vs. open/hybrid 38.6 ± 21, *p* = 0.013). After revascularization, mean perfusion values did not significantly differ between the two treatment groups. When analyzing the increase in perfusion after revascularization according to revascularization technique, StO2 was the only perfusion parameter significantly higher in the endovascular group compared with the hybrid/open surgical group (endo +8 ± 16.5 vs. hybrid/open +1.7 ± 14.7, *p* = 0.044).

Furthermore, the influence of the Rutherford category before the revascularization on the perfusion parameters was analyzed. In CLTI patients, the TcPO2 and ABI values were significantly lower than in Rutherford category 3 patients before the revascularization (TcPO2 28.4 ± 23.6 vs. 47.5 ± 19.8, *p* < 0.001; ABI 0.4 ± 0.16 vs. 0.52 ± 0.16, *p* = 0.003). Of all HSI parameters, THI was the only parameter demonstrating a significant pre-revascularization difference between CLTI and non-CLTI patients, with higher mean values in CLTI patients compared to Rutherford class 3 patients (CLTI 27 ± 14.3 vs. non-CLTI 20.5 ± 11, *p* = 0.027). After the revascularization, mean ABI values were significantly lower in the CLTI group (0.74 ± 0.23 vs. 0.87 ± 0.21, *p* = 0.006); however, TcPO2 values were equal between the two groups (57.9 ± 31.5 vs. 57.7 ± 22.7). Of the HSI parameters, StO2 was significantly higher in the CLTI cohort after revascularization compared to patients with claudication (51.3 ± 12.6 vs. 43.9 ± 12.4, *p* = 0.003). THI and NIR were not significantly different between the two groups.

### 3.2. Clinical Outcome

**Figure 4:**
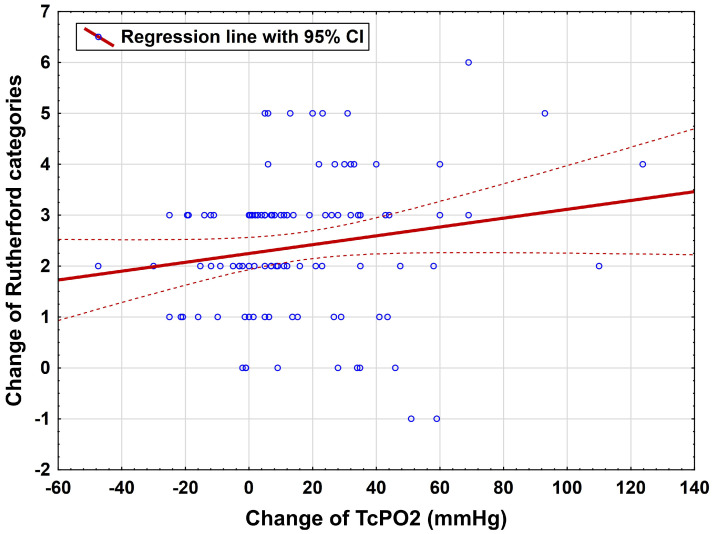
Correlation of Rutherford category improvement with TcPO2 measurements, n = 103; TcPO2: transcutaneous oxygen pressure measurement. The dotted line is the 95 CI as indicated in the left upper corner.

Most patients (n = 56, 54%) presented with an asymptomatic clinical status followed by Rutherford category 1 (n = 24, 23%) at the three-month follow-up. Four patients suffered from acute occlusion of their femoropopliteal bypass, and one occlusion of an iliac stent was detected during the three-month follow-up period. A significant correlation between the improvement of TcPO2 and the improvement of Rutherford categories could be detected after revascularization (r = 0.20, 95% CI: 0.005–0.38, *p* = 0.046, Figure 4). However, no such correlation was seen between the three HSI parameters and the Rutherford categories (StO2 *p* = 0.83, THI *p* = 0.94, NIR *p* = 0.65).

Ten out of the twelve patients with tissue loss before revascularization achieved wound healing within three months. In the remaining two patients, a considerable improvement in wound healing could be achieved. No unplanned minor or major amputation was necessary. The mean TcPO2 value in patients with Rutherford categories 5 and 6 was 23 ± 22.3 mmHg prior to revascularization, corresponding to ischemia grade 3 in the WIfI classification. After revascularization, the mean TcPO2 increased to 55 ± 36 mmHg, corresponding to ischemia grade 1. The two patients without complete wound healing at 3 months had TcPO2 values of 54 and 49 mmHg, respectively, both consistent with ischemia grade 1. Regarding the HSI measurements of these 12 patients, again, a high number of worse parameters despite successful revascularization was detected. Five (42%) StO2, eight (67%) THI and six (50%) NIR measurements were stable or worse after revascularization.

## 4. Discussion

In this prospective study, the ability of HSI to assess tissue perfusion after successful revascularization in correlation with TcPO2 measurements and clinical results was evaluated. A statistically significant improvement of TcPO2, NIR and StO2 values could be detected, while THI values showed no improvement. There was a high number of worse or stable HSI results despite clinically successful revascularization. A significant correlation of ABI with TcPO2 and NIR was detected, which was not the case for TcPO2 or ABI with StO2 and THI. Regarding the clinical outcomes, again TcPO2 but not HSI parameters showed a significant correlation with the change of Rutherford categories during follow-up.

Chiang et al. presented one of the largest studies utilizing HSI for the assessment of PAD severity in correlation to TcPO2 and ABI (n = 294, including healthy volunteers). With their HSI device (OxyVu, HyperMed Inc, Burlington, MA, USA), the oxygen saturation (HT-Sat) as well as the density of oxy- and deoxyhemoglobin was measured. The study concluded good reliability of the described measurements and low inter- and intraoperator variability [9]. However, their claimed reliability was based on only modest correlation parameters, and therefore, the clinical significance remains unclear. The TIVITA system used in our study, unfortunately, does not provide information on oxy- and deoxyhemoglobin levels. The good correlation of HT-Sat with ABI and TcPO2 of Chiang et al. cannot be confirmed in our study. The composition of their and our study cohorts differed in terms of Rutherford category distribution. Our cohort consisted of 31% CLTI patients, whereas Chiang et al. had a rate of 77%. Despite the high number of Rutherford 3 patients in our cohort, the median TcPO2 value of this cohort was 50 (IQR 38.4–57.7) mmHg, reflecting their impaired lower extremity tissue perfusion. After the revascularization, a significant improvement of TcPO2 was also detected in the Rutherford 3 cohort; therefore, one would expect improved HSI values accordingly. This indicates that, irrespective of the differing CLTI proportions in our and Chiang’s cohorts, the extent of compromised tissue perfusion was similar.

Grambow et al. compared StO2 and NIR values of healthy individuals and PAD patients with the TIVITA^®^ camera system. In this study, the two HSI parameters StO2 and NIR perfusion index, were significantly lower in PAD patients compared to the healthy cohort. However, skin temperature was not significantly different, and no other perfusion measurement methods were applied. They concluded good correlation between NIR perfusion index and StO2 to ABI, complaint-free walk distance and vascular quality of life score. However, only the NIR perfusion index showed a significant correlation to the aforementioned parameters, whereas the correlation of StO2 was not statistically significant. Furthermore, ABI measurement was not feasible in 37% of PAD patients, and due to the small cohort (25 patients per group), the study findings warrant cautious interpretation [14]. In another study by Grambow et al., HSI measurements were performed before and after revascularization for PAD in a small cohort of 37 patients with a comparable study protocol to ours. They stated that StO2 and NIR perfusion index showed significant changes after perfusion improvement in almost all angiosomes and that HSI is a valuable alternative to monitor PAD. However, three days after surgery, no significant StO_2_ improvement was observed in any angiosome, and after endovascular revascularization, only two out of seven angiosomes showed measurable improvement. The NIR perfusion index significantly improved in five of seven angiosomes in the surgical group, whereas only two significant improvements were detected in the endovascular group. Consequently, the authors’ conclusion should be viewed with some reservation. In our study, the unexpectedly high proportion of stable or deteriorating HSI values despite technically successful revascularization was noteworthy. This observation calls the reliability of the remaining measurement parameters into question. Unfortunately, Grambow et al. provided no information about worse or stable HSI parameters after revascularization in their cohort [8]. As outlined in our results, worse measurements after revascularization were noted for TcPO2, but more pronounced for HSI parameters. The most likely reasons for a decrease in TcPO2 after revascularization could be postoperative edema or microvascular disease of the treated leg. However, the swelling can persist up to 2–4 weeks after the revascularization. Therefore, perfusion assessment, independent of the method applied, can be challenging. HSI measurement also depends on the penetration depth of the emitted laser light. HSI and TcPO2 measurements were obtained on the same day; therefore, HSI results may be influenced to the same extent by edema. However, as shown in our results, the HSI parameters were markedly less reliable.

Especially in patients with tissue loss due to PAD or diabetes, reliable diagnostic methods for the evaluation of the microcirculation are pivotal. Lopéz-Moral et al. investigated tissue perfusion in patients with diabetic foot ulcers with TcPO2 and HSI. They identified an StO2 threshold with high sensitivity and specificity for predicting ulcer healing, whereas THI and NIR showed no significant associations. Their logistic regression analysis identified TcPO2, but no HSI parameter, as the only diagnostic method reliably predicting wound healing at 24 weeks [16]. This goes in line with our results, as, in contrast to TcPO2, no HSI parameter demonstrated a significant correlation with clinical improvement after revascularization. Furthermore, TcPO2 and ABI were significantly lower in the CLTI cohort compared to the Rutherford 3 group; however, none of the HSI values showed similar results. Interestingly, after the revascularization, ABI values stayed significantly lower in the CLTI cohort; however, the TcPO2 values did not significantly differ between the Rutherford categories. Whether these results were based on the clinical stage of the patients or were also influenced by the revascularization strategy remains unclear. Notably, the hybrid/open surgery group included significantly more patients with CLTI than the endovascular group. However, this distribution reflects real-world practice. Many CLTI patients underwent prior revascularization and often presented with more complex lesions, leading to a higher number of surgically treated CLTI patients.

Two systematic reviews of Saiko et al. and Yli-Harja et al. summarize the role of HSI for the diagnosis and evaluation of the healing potential of wounds and PAD in general [12,13]. Both reviews outline one of the biggest limitations of the studies investigating the role of HSI in PAD and wound diagnostics. There are different manufacturers of HSI devices with different software providing several parameters. Furthermore, no uniform nomenclature for the same parameters is used, impeding comparability even more. Another important difference between different manufacturers is the measured and evaluated wavelength range of the camera system. As absorption and scattering of emitted light influence different wavelengths to varying degrees, measurements at only a few wavelengths are insufficient to identify the underlying cause of signal changes, requiring additional light characteristics to be considered. Considering the aforementioned factors, comparison across HSI studies is limited by the heterogeneity of analyzed and reported parameters as well as technical reasons. Khaodhiar et al., like the aforementioned study of Chiang et al., analyzed oxy- and deoxyhemoglobin values in the surrounding area of diabetic foot ulcers. They demonstrated that these two parameters have the capability of predicting wound healing in foot ulcers [17]. Ubbink et al., on the other hand, examined the reproducibility of spectroscopy for the diagnostic workup of leg ischemia, but only one HSI parameter was evaluated. The reproducibility of the tissue oxygenation was excellent; however, no correlation of this parameter to other micro- and macrocirculatory parameters could be observed [18]. Similar results were seen in our study, with no correlation between TcPO2 and the HSI parameters. Of the three HSI parameters investigated, only the NIR showed a significant correlation with ABI.

### Limitations of This Study

This is a prospective cohort study; however, patients with different clinical symptoms ranging from Rutherford categories three to six were included. The evaluation of tissue perfusion in a mixed cohort of intermittent claudication and CLTI patients may limit the validity of our results. Nevertheless, our TcPO2 measurements and the improvement after revascularization suggested impaired tissue perfusion and the potential for improvement also in the Rutherford 3 cohort. Furthermore, the revascularization strategies included endovascular and open surgical as well as hybrid procedures, maybe also limiting the validity of the results. The results of our study were obtained with a device from a single HSI manufacturer; therefore, limited generalizability of our results due to manufacturer-specific technical and analytical characteristics might be given. The single-center design of this study, the absence of operator blinding to clinical status, and the lack of intra- and interobserver variability analyses for HSI constitute further limitations of this study.

## 5. Conclusions

Significant improvement of StO2, NIR and TcPO2 values was detected after successful revascularization; however, no correlation of HSI parameters with TcPO2 or clinical results could be observed. Furthermore, the substantial rate of lower or stable HSI parameters despite clinical improvement and increased TcPO2 values calls the validity and clinical relevance of TIVITA^®^-based HSI measurements as a quality control of perfusion improvements after revascularization into question. Our data suggests that TcPO2 remains the clinical standard for the evaluation of tissue perfusion in PAD patients. Due to the conflicting TIVITA^®^-based HSI results, further research is needed to determine its clinical value.

## Figures and Tables

**Table 1:** Demographics of the patients (n = 103).

	Number (%)
Age	69 (63–76) ^§^
Male	68 (66) *
CABG	11 (10.7) *
CAD	47 (45.6) *
Diabetes mellitus	36 (35) *
Dyslipidemia	58 (56.3) *
Hypertension	79 (76.7) *
Smoker current	45 (43.7) *
Smoker former	28 (27.2) *
PCI	26 (25.2) *
Acetylsalcylic acid	77 (74.8) *
Clopidogrel	29 (28.2) *
Vitamin K antagonists	7 (6.8) *
DOAK	16 (15.5) *
Statin	90 (87.4) *
Metformin	17 (16.5) *
Rutherford category 3	71 (68.9) *
Rutherford category 4	20 (19.4) *
Rutherford category 5	10 (9.7) *
Rutherford category 6	2 (1.9) *
WIfI stage 1	1 (1%)
WIfI stage 3	5 (4.9%)
WIfI stage 4	6 (5.8%)
ABI pre-revascularization	0.5 (0.4–0.6) ^§^
TcPO2 pre-revascularization	43 (26–57) ^§^

^§^ median (interquartile range) * absolute numbers (%), ABI: ankle–brachial index, CAD: coronary artery disease, CABG: coronary artery bypass graft, DOAK: direct oral anticoagulation, PCI: percutaneous coronary intervention, WIfI: wound, ischemia, foot infection classification.

## Data Availability

The data presented in this study are available on request from the corresponding author.

## Abbreviations

The following abbreviations are used in this manuscript:

ABI	Ankle–brachial index
CABG	Coronary artery bypass graft
CLTI	Chronic limb-threatening ischemia
DOAK	Direct oral anticoagulation
HSI	Hyperspectral imaging
NIR	Near-infrared
PAD	Peripheral arterial disease
PCI	Percutaneous coronary intervention
StO2	Tissue oxygenation
TcPO2	Transcutaneous oxygen pressure measurement
THI	Tissue hemoglobin index
